# Rapamycin-independent *IGF2* expression in *Tsc2*-null mouse embryo fibroblasts and human lymphangioleiomyomatosis cells

**DOI:** 10.1371/journal.pone.0197105

**Published:** 2018-05-14

**Authors:** Blanca E. Himes, Kseniya Obraztsova, Lurong Lian, Maya Shumyatcher, Ryan Rue, Elena N. Atochina-Vasserman, Stella K. Hur, Marisa S. Bartolomei, Jilly F. Evans, Vera P. Krymskaya

**Affiliations:** 1 Department of Biostatistics, Epidemiology and Informatics, Perelman School of Medicine, University of Pennsylvania, Philadelphia, Pennsylvania, United States of America; 2 Penn Center for Pulmonary Biology and Pulmonary, Allergy and Critical Care Division, Perelman School of Medicine, University of Pennsylvania, Philadelphia, Pennsylvania, United States of America; 3 Epigenetics Institute and Department of Cell and Developmental Biology, Perelman School of Medicine, University of Pennsylvania, Philadelphia, Pennsylvania, United States of America; Medical University of South Carolina, UNITED STATES

## Abstract

Lymphangioleiomyomatosis (LAM) is a rare, almost exclusively female lung disease linked to inactivating mutations in *tuberous sclerosis complex 2* (*TSC2)*, a tumor suppressor gene that controls cell metabolic state and growth via regulation of the mechanistic target of rapamycin (mTORC1) signaling. mTORC1 is frequently activated in human cancers and, although the mTORC1 inhibitor rapamycin has a cytostatic effect, it is, in general, unable to elicit a robust curative effect or tumor regression. Using RNA-Seq, we identified (1) *Insulin-like Growth Factor* (*IGF2*) as one of the genes with the highest fold-change difference between human *TSC*2-null and *TSC*2-expressing angiomyolipoma cells from a patient with LAM, and (2) the mouse *IGF2* homolog *Igf2*, as a top-ranking gene according to fold change between *Tsc*2^-/-^ and *Tsc*2^+/+^ mouse embryo fibroblasts (MEFs). We extended transcript-level findings to protein level, observing increased Igf2 protein expression and Igf2 secretion by *Tsc*2^-/-^ MEFs. Increased Igf2 expression was not due to epigenetic imprinting, but was partially mediated through the Stat3 pathway and was completely insensitive to rapamycin treatment. An siRNA-mediated decrease of Igf2 resulted in decreased Stat3 phosphorylation, suggesting presence of an autocrine Igf2/Stat3 amplification cycle in *Tsc2*^*-/-*^ MEFs. In human pulmonary LAM lesions and metastatic cell clusters, high levels of IGF2 were associated with mTORC1 activation. In addition, treatment of three primary IGF2-expressing LAM lung cell lines with rapamycin did not result in IGF2 level changes. Thus, targeting of IGF2 signaling may be of therapeutic value to LAM patients, particularly those who are unresponsive to rapamycin.

## Introduction

The mechanistic target of rapamycin (mTORC1) is a central controller of cell growth and metabolism [1]. mTORC1 is frequently activated in human cancers due to mutational activation of oncogenes or inactivation of tumor suppressors, including the *tuberous sclerosis complex 2* (*TSC2*) gene [2]. Pulmonary lymphangioleiomyomatosis (LAM), a rare progressive lung disease affecting primarily women of childbearing age that is characterized by cyst rapture, chylothorax from obstruction of lymphatics, and progressive decline of pulmonary function, has also been associated with inactivating mutations and loss of function of *TSC2* that lead to uncontrolled mTORC1 activation and cell growth [3–6]. There are two forms of LAM: one that is associated with tuberous sclerosis complex (LAM-TS), in which women carry germline *TSC2* mutations, and sporadic LAM (LAM-S), in which *TSC2* mutations and loss of heterozygosity arise in somatic tissues post-conception [3]. Approximately 80% of LAM-TS and approximately 40% of LAM-S patients also develop angiomyolipoma (AML), a benign tumor of smooth muscle (SM), blood vessels and fat cells, usually occurring in the kidney [7].

Rapamycin (sirolimus), an allosteric inhibitor of the mTOR complex [8], is currently the only FDA-approved drug for LAM. Benefits of its use were demonstrated by an international two-stage, double-blinded clinical trial among LAM patients with moderate lung impairment in which those taking the drug had stabilized lung function and improved quality of life [9, 10]. Unfortunately, rapamycin only has a cytostatic effect on tumor growth [11] and requires life-long treatment with considerable side-effects [12]. Because no other treatments are available, there is an urgent need to discover new LAM drug targets.

Insulin-like Growth Factor (IGF2), a small polypeptide closely related in sequence and structure to insulin, is a key growth regulator in some dominantly female proliferative diseases that activates multiple pathways involved in cell proliferation, growth and survival [13, 14]. In addition to being involved in breast development and cancer, and in colon, ovarian, prostate and fibrous sarcomas [13], IGF2 has been associated with LAM, as immunohistochemical studies found that IGF2 was expressed in the cytoplasm and surface of spindle-shaped LAM lung cells [15]. We show here that IGF2 is expressed in TSC2-null mouse embryo fibroblasts (MEFs) and in human LAM cells, but it is insensitive to rapamycin treatment, and thus, targeting its signaling pathway is a potentially novel LAM therapeutic avenue.

## Materials and methods

### Ethics statement

De-identified lung tissue samples from patients with advanced LAM disease who had undergone lung transplantation and healthy controls were received from the National Disease Research Interchange (NDRI) in compliance with University of Pennsylvania Institutional Review Board-approved procedures. Use of these tissues does not constitute human subjects research since all donor tissue is harvested anonymously and de-identified.

### Cell cultures

*Tsc2*^*-/-*^ mouse embryo fibroblasts (MEFs) and wild type *Tsc2*^*+/+*^ MEFs were generously provided to us by Dr. David Kwiatkowski, Brigham and Women’s Hospital [16]. Human TSC2-null 621–102 LAM (TSC2—) cells and TSC2 re-expressing 621–103 LAM (TSC2++) cells [17] were derived from angiomyolipoma of patient with sporadic LAM and obtained via a generous gift from Dr. Lisa Henske, Brigham and Women’s Hospital. The LAM-patient TSC2— and TSC2++ cells were genetically characterized for *TSC2* loss-of-function mutation and expression of estrogen receptor [17]. Primary human LAM cells were derived from LAM tissue as described previously [4]. Cells were maintained in DMEM (Gibco) supplemented with 10% FBS. For RNA-Seq Experiment, cell lines were treated with either (1) 20nM STAT3 siRNA (Dharmacon) for 48 hr, or (2) 20nM non-targeting (NT) siRNA for 48 hr in complete medium.

### RNA-seq library construction and sequencing

Total RNA was extracted using the miRNAeasy mini kit (Qiagen Sciences, Inc., Germantown, MD). The Illumina TruSeq Stranded mRNA LT Sample Prep Kit (Illumina, Inc., San Diego, CA) was used to prepare poly(A)-selected stranded RNA-Seq libraries, and 100bp paired-end reads were generated with an Illumina Hi-Seq 2500 instrument in high output mode.

### RNA-Seq data analysis

RNA-Seq reads were trimmed with Trimmomatic (v.0.32). Subsequently, Kallisto was used to quantify transcript abundance, using the hg38 human transcriptome as reference [18]. R was used to compute fold changes based on these results, adding 1 to all TPM values to avoid division by zero [19]. The NIH Database for Annotation, Visualization and Integrated Discovery (DAVID) was used to perform gene ontological category enrichment analysis using Homo Sapiens as background, and default options and annotation categories (Disease: OMIM_DISEASE; Functional Categories: COG_ONTOLOGY, SP_PIR_KEYWORDS, UP_SEQ_FEATURE; Gene_Ontology: GOTERM_BP_FAT, GOTERM_CC_FAT, GOTERM_MF_FAT; Pathway: BBID, BIOCARTA, KEGG_PATHWAY; Protein_Domains: INTERPRO, PIR_SUPERFAMILY, SMART) [20]. Trimmed reads were aligned to the hg38 genome using STAR (v. 2.5.2a) [21], and aligned reads were used to create mapped read plots by displaying bigwig files for each sample in the UCSC Genome Browser. RNA-Seq data is available in the Gene Expression Omnibus (GEO) under series accession number GSE84478, with human samples in subseries GSE84476 and mouse samples in subseries GSE84477.

### Genomic imprinting

Genomic DNA samples were isolated from primary human LAM cell lines and normal human lung fibroblast cells. For DNA isolation, cells were lysed in 0.5% SDS-containing TE buffer (pH 8.0) followed by Proteinase K treatment at 55°C overnight. Genomic DNA was then isolated using standard phenol-chloroform method and re-suspended in TE buffer. One microgram of each DNA sample was bisulfite-treated using the EpiTect Bisulfite Kit (Qiagen Sciences, Inc., Germantown, MD) following manufacturer's protocol. Pyrosequencing was performed to analyze the methylation profiles at the *H19/IGF2* imprinting control region (ICR) as described previously [21]. 50ng of bisulfite-treated DNA was used for PCR. The PyroMark PCR kit (Qiagen Sciences, Inc., Germantown, MD) was used in a 25μL reaction according to the manufacturer's protocol. PCR conditions were: 95°C for 15 minutes followed by 45 cycles of 95°C for 30 seconds, 55° for 30 seconds and 72°C for 30 seconds, and 5 minutes of extension at 72°C. 10μL of the biotinylated PCR product was used for pyrosequencing. Pyrosequencing was done using the PyroMark Q96MD (Qiagen Sciences, Inc., Germantown, MD) system following the manufacturer's protocol and the PyroMark Gold reagents kit (Qiagen Sciences, Inc., Germantown, MD). Methylation was analyzed on 6 CpGs using Qiagen's Pyro Q-CpG software [22].

### Chromatin immunoprecipitation (ChIP)-qPCR

Chromatin immunoprecipitation (ChIP) was performed on *Tsc2*^+/+^ and *Tsc2*^-/-^ MEFs and human TSC2— and TSC2++ cells using the Magna ChIP kit (Millipore, Burlington, MA) and monoclonal anti-STAT3 antibody (Millipore, Burlington, MA) as described https://www.protocols.io/private/12361d13ea65a41e2d2a98f529d6a745. Input and IP DNA samples were purified using ChIP DNA Clean and Concentrator kit (Zymo Research, Irvine, CA). qPCR analysis of chromatin pull-down was performed using primers amplifying regions containing STAT3 binding sites within *IGF2* promoters. The amounts were normalized to the level of input chromatin material prior to immunoprecipitation. Reproducibility between biological replicates was statistically assessed using 2-way ANOVA. The following DNA primers were used: Mouse *Igf2*_P2_Forward: GGCCCCATAATTTAGGAACCCA; Mouse *Igf2*_P2_Reverse: TTTGGAGTACCTGAATTTGGGGG. Mouse *Igf2*_P5_Forward: AAGAGTCAAGCCAGACCCCA; Mouse *Igf2*_P5_reverse: ATTTCTGCCCTTCTGAGCCC. Human *IGF2*_P2 forward: GCCATTTTACCAGTGCCACG; Human *IGF2*_P2_reverse: CTAGGAGGTGGGGGCTATGT. Human *IGF2*_P4_Fw: CTAGCGTTGCCCAAACACAC; Human IGF2_P4_reverse: CCCAGTCCGTTGGAAGACC.

### Immunoblots

For protein extraction, cultured cells were lysed in RIPA lysis buffer (Cell Signaling, Danvers, MA) with freshly added PMSF, sodium orthovanadate and complete protease inhibitors [23]. Protein concentration was determined with the Pierce^TM^ BCA protein assay (ThermoFisher, Philadelphia, PA). Immunoblotting was done with anti-IGF2 antibody (Biorbyt, Cambridge, UK) according to the manufacturer’s protocol. Briefly, 4–12% Bis-Tris polyacrylamide gels (NuPage, Invitrogen) were equally loaded with 5–30μg of protein, electrophoresed at 140-200V, and transferred to nitrocellulose membrane by iBlot dry transfer. Membranes were blocked with either 5% BSA or LI-COR TBS blocking buffer for 30 minutes to 1 hr. The membrane was probed with primary antibodies to IGF2 (Biorbyt, Cambridge, UK) and pSTAT3, STAT3, TSC2, pS6, S6, GAPDH and β-actin (all from Cell Signaling Technology, Inc., Beverly, MA) at 4°C overnight. Appropriate secondary antibodies were incubated for 1 hr at room temperature. Blots were developed either by ECL prime Western blotting detection reagents (GE—Amersham Bioscience, Piscataway, NJ) or by secondary fluorescent antibodies (LI-COR, Lincoln, NE). Images were captured using a LI-COR Odyssey-Fc imager with LI-COR advanced imaging software.

### Enzyme-Linked ImmunoSorbent Assay (ELISA)

Cultured media was collected following centrifugation at 4°C at 15,000g for 15 min. IGF2 concentration was determined using a mouse IGF2 ELISA kit (Biorbyt, Cambridge, UK) according to the manufacturer’s protocol.

### Immunohistochemistry (IHC)

IHC analysis of formalin-fixed paraffin-embedded control or LAM lung tissue was performed using specific antibodies against human IGF2 (Biorbyt, Cambridge, UK), phospho-ribosomal protein S6 (pS6, Cell Signaling Technology, Inc., Beverly, MA), and SM a-actin (Sigma Chemical Co., St. Louis, MO). Staining was performed on a Leica Bond™ instrument using the Bond Polymer Refine Detection System (Leica Microsystems, Buffalo Grove, IL). Stained slides were scanned to create digital whole slide images (WSI) using an Aperio ScanScope XT (Aperio Technologies, Vista, CA) with 20x magnification (0.46 μm per pixel). Data from each slide was automatically captured and stored in the Aperio Spectrum v11 database of the Pathology Clinical Service Center at the University of Pennsylvania. Each WSI was analyzed using Aperio ImageScope software (http://www.aperio.com/#imagescope-request).

## Results

### Transcriptomic differences in response to TSC2 loss

**Fig 1:**
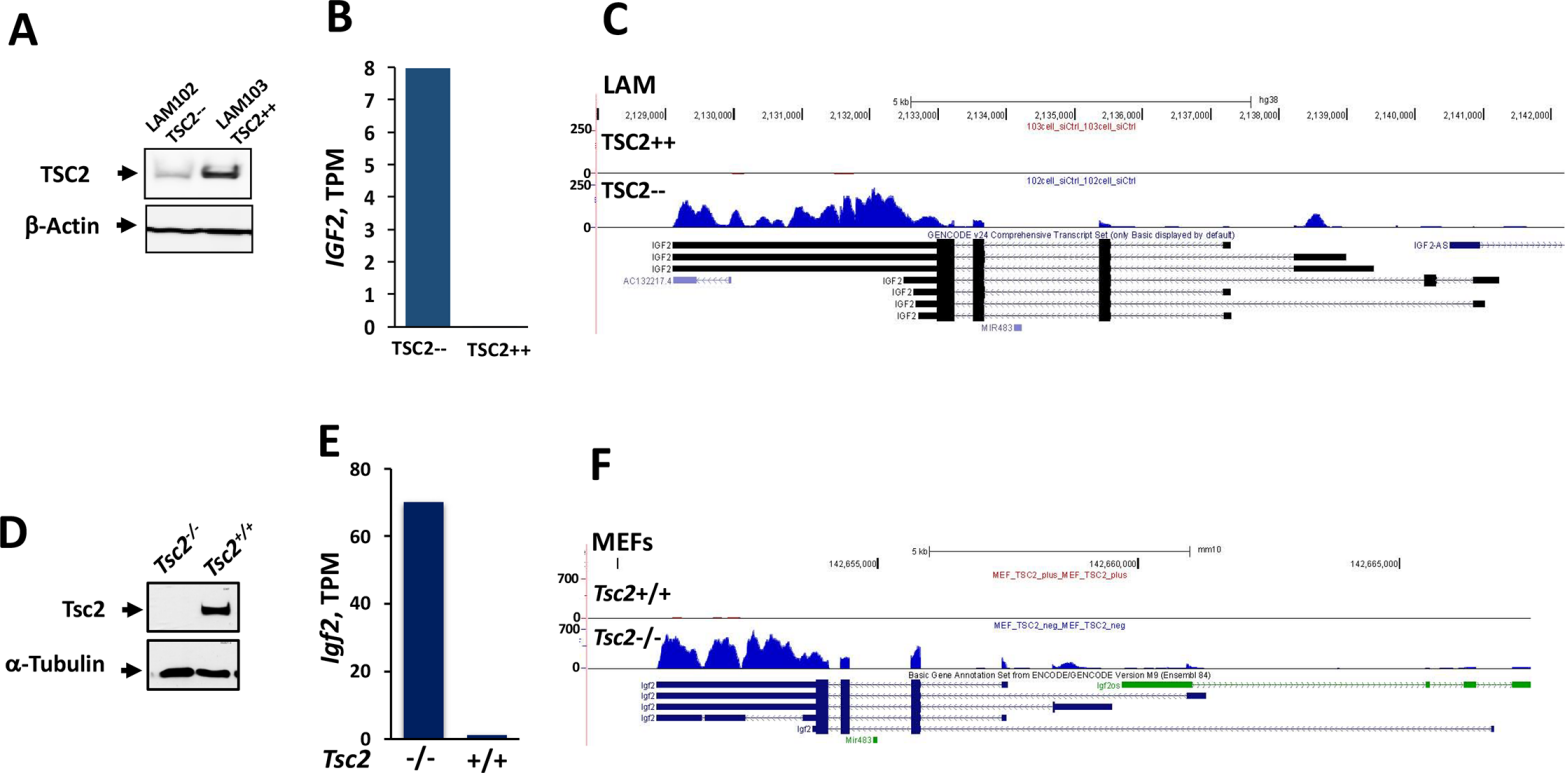
Increased expression of *IGF2* transcripts in TSC2— human LAM cells and *Tsc2*^*-/-*^ MEFs. (A) TSC2 levels in human TSC2-null LAM 621–102 cells (TSC2—) cells and TSC2 re-expressing 621–103 LAM (TSC2++) cells. (B) RNA-Seq results show increased *IGF2* transcripts per kilobase million (TPM) in TSC2— cells. (C) Corresponding plot of mapped reads along the hg38 reference genome corresponding to *IGF2*. (D) Verification that *Tsc2* is not expressed in *Tsc2*^*-/-*^ MEFs. (E) RNA-Seq results show increased *Igf2* TPMs in *Tsc2*^*-/-*^ vs. *Tsc2*^*+/+*^ MEFs. (F) Corresponding plot of mapped reads along the mm10 reference genome corresponding to *Igf2*.

RNA-Seq was performed using human *TSC2*-null 621–102 LAM (TSC2—) cells, which carry G1832A missense mutation in exon 16 in one allele [17], and TSC2 re-expressing 621–103 LAM (TSC2++) cells verified to re-express *TSC2* (Fig 1A). Six human samples were analyzed: TSC2— and TSC2++ cells that were either (1) transfected with STAT3 siRNA, or (2) treated with non-targeting (NT) siRNA. Additionally, two mouse samples were compared, *Tsc2*^*-/-*^ MEFs, which were verified to not express *Tsc2* due to genetic deletion, and wild-type *Tsc2*^*+/+*^ MEFs, which were verified to express *Tsc2* (Fig 1D). After quality control processing, each of the 8 samples were deemed of sufficiently high quality to include in further analyses (S1 Table). Comparison of *Tsc2*^*-/-*^ and *Tsc2*^*+/+*^ MEFs’ transcript expression levels found that 732 transcripts, corresponding to 621 unique genes, had an absolute fold change ≥ 8 (S2 Table). Comparison of transcript expression levels (as transcripts per kilobase million reads–TPM) between human TSC2— and TSC2++ cells treated with NT siRNA found that 1137 transcripts, corresponding to 768 unique genes, had an absolute fold change ≥ 8 (S3 Table). To gain a better sense of the biological functions represented by top-ranked genes from the mouse and human comparisons, we performed an ontological category enrichment analysis using the NIH DAVID tool [20]. There were 12 and 7 annotation clusters with enrichment scores >2 for the mouse and human comparisons, respectively (S4 and S5 Tables). Among these clusters, some of the individual enrichment categories had Benjamini-Hochberg adjusted p-values < 0.05 and referred to processes that are associated with LAM, namely *signaling* and *insulin-like growth factor binding*.

Genes were prioritized for further experimental study by (1) selecting genes with high absolute fold change in the mouse comparison, (2) selecting the subset of these genes with high absolute fold change in human comparison, and (3) favoring genes in LAM-related ontological categories. Among highly ranked genes was *IGF2*, which was present in TSC2— cells (8.0 TPM) but not appreciably expressed in TSC2++ cells (S3 Table and Fig 1B). Consistent with the results in human cells, the *Tsc2*^*-/-*^ vs. *Tsc2*^*+/+*^ MEFs comparison found that *Igf2* was present in *Tsc2*^*-/-*^ cells (69.3 TPM) but not appreciably expressed in *Tsc2*^*+/+*^ cells (S2 Table and Fig 1E). Plots of mapped human reads in/near *IGF2* along the hg38 reference genome and mouse reads in/near *Igf2* along the mm10 reference genome show appropriate coverage of each gene (Fig 1C and 1F). *IGF2*/*Igf2* was part of the over-represented *signaling pathways* ontological category (S4 and S5 Tables).

### IGF2 expression in LAM lung

**Fig 2:**
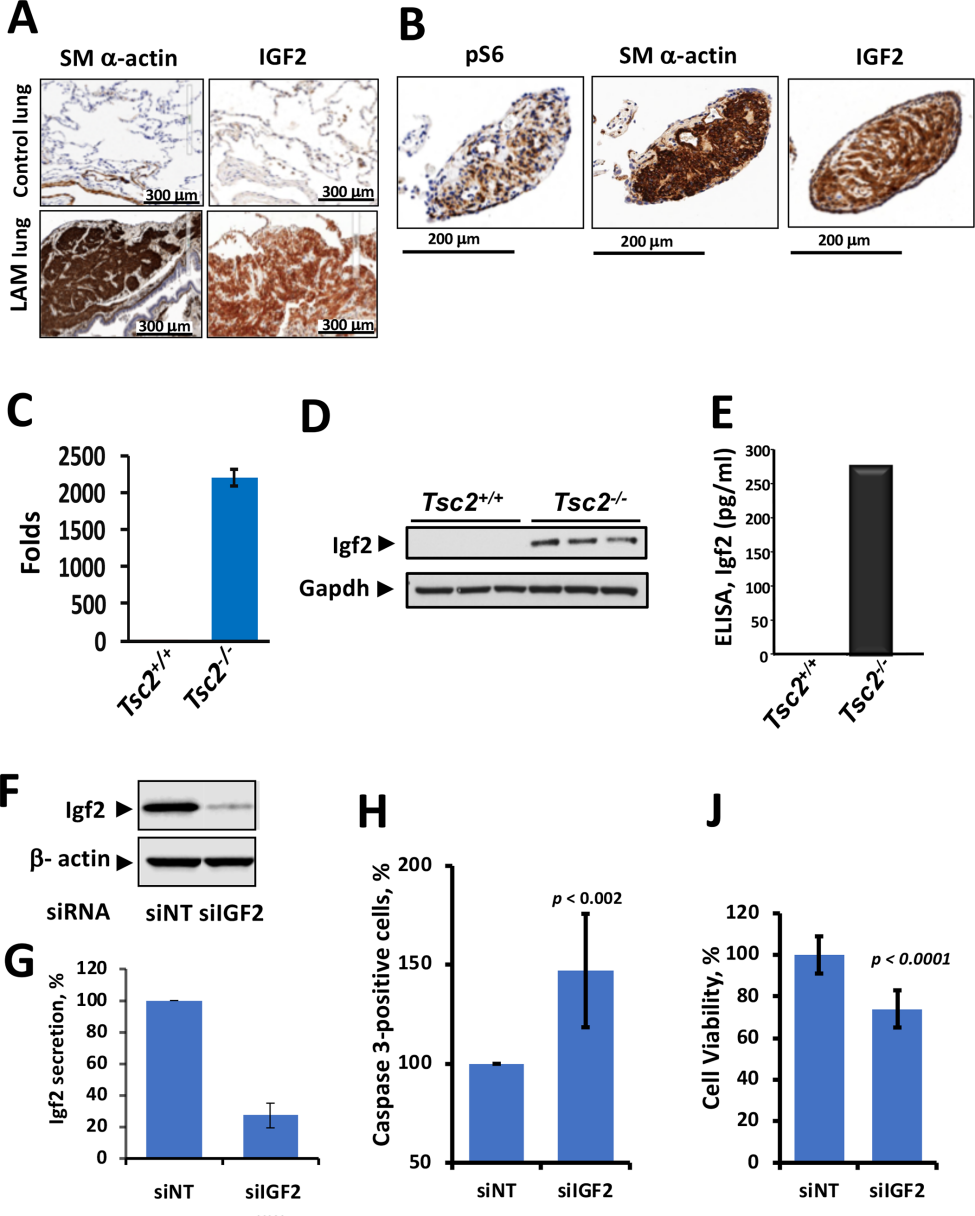
IGF2 expression in LAM lungs and *Tsc2*^*-/-*^ MEFs. Representative images of IHC analysis show IGF2 expression in (A) LAM lesion and (B) LAM cluster detected with specific antibodies. Non-immune IgG was used as a control (see S1 Fig). Igf2 expression in *Tsc2*^*-/-*^ MEFs was detected by (C) qPCR (D) Western blot and (E) ELISA. (F) *Tsc2*^*-/-*^ MEFs were transfected with 50nM *Igf2* siRNA (siIGF2) or NT siRNA (siNT) for 48 hrs. Decreased levels of Igf2 protein expression were confirmed via western blot with β-actin as an internal loading control. (G) Decreased Igf2 protein secretion was confirmed via ELISA. Igf2 knockdown resulted in (H) increased cleaved caspase-3 levels as measured via immunocytostaining and flow for Alexa Fluor® 488 -Cleaved Caspase 3 where the population of positively stained MEFs was normalized to the control population, and (J) decreased cell viability as assessed by 0.4% Trypan Blue staining normalized to the control cell viability. Student's t-tests were used to determine the statistical significance of the differences, and *p*-values reflect a sample size of 3 replicates.

We used human lung LAM tissues from transplant donors with advanced LAM disease to detect whether IGF2 is expressed *in vivo*. LAM lung lesions, which were detected using the LAM cell marker SM a-actin, showed marked IGF2 reactivity compared to normal lungs (Fig 2A and S1 Fig). IGF2 immunoreactivity was also detected in LAM clusters, which are LAM lesions that disseminate via lymphatics [24] (Fig 2B). LAM clusters also expressed SM a-actin and pS6, two positive control markers of LAM clusters (Fig 2B). These data are consistent with previous reports showing that IGF2 is upregulated specifically in LAM cells [15].

### Igf2 protein expression and secretion is induced by *TSC2* loss in MEFs

To validate transcriptomic data, we measured *Igf2* transcript expression using qRT-PCR, and to extend transcriptomic results to the level of protein, we measured Igf2 protein expression using immunoblots and secretion using ELISAs. In *Tsc2*^*-/-*^ MEFs *Igf2* transcripts and Igf2 protein levels were highly increased compared to undetectable levels in *Tsc2*^*+/+*^ MEFs (Fig 2C and 2D). *Tsc2*^*-/-*^ MEFs also secreted marked levels of Igf2 compared to *Tsc2*^*+/+*^ MEFs (Fig 2E). Downregulation of *Igf2* with siRNA (Fig 2F), significantly decreased Igf2 secretion (Fig 2G). *Igf2* siRNA-mediated knockdown significantly increased the number of cleaved caspase-3 positive cells (Fig 2H) and decreased *Tsc2*^*-/-*^ MEF viability (Fig 2J). Thus, *Igf2* transcript expression changes extended to the protein level, and Igf2 protein expression increased *Tsc2*^*-/-*^ MEF survival.

### Epigenetic imprinting of *IGF2* in LAM

Previous reports have found that *IGF2* gene dosage is modified via genomic imprinting, achieved either through loss of repression of the maternal allele, resulting in biallelic expression of the gene, or through de-regulated expression of *IGF2* from the paternal allele [25, 26]. To determine whether genomic imprinting contributes to differences in *IGF2* expression observed in LAM cells, we measured DNA methylation levels at the *IGF2* gene imprinting control region 1 (IC1) in female human LAM cells, female *TSC2*-null 621–102 LAM (TSC2—) cells, TSC2 re-expressing 621–103 LAM (TSC2++) cells as well as healthy human airway smooth muscle (ASM) cells derived from male and female donors as controls. The IC1 was methylated near 50%, indicative of normal imprinting status, in all cell lines except for in TSC2— LAM cells (S6 Table). The lower IC1 methylation level (23%) measured in these cells, however, is predictive of reduced IGF2 RNA levels [26]. De-regulated expression of paternal IGF2 is therefore the likely reason for elevated IGF2 levels in TSC2— LAM cells.

### IGF2 expression in TSC2-null cells is STAT3-mediated

**Fig 3:**
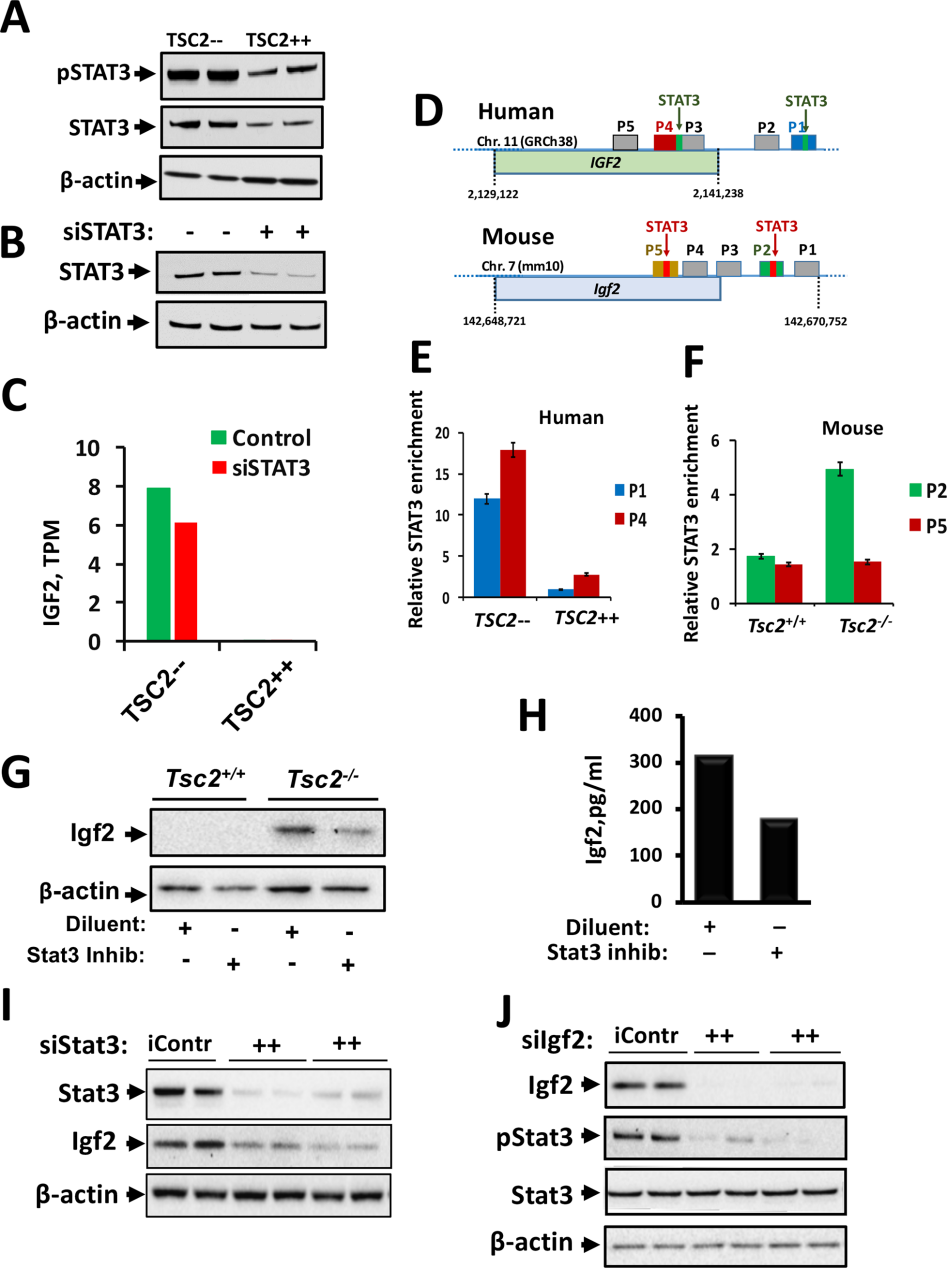
STAT3-dependent upregulation of IGF2 in TSC2-null cells. (A) Re-expression of TSC2 (TSC2++) in TSC2-null LAM 102 (TSC2—) cells decreased STAT3 expression and activation. (B) siRNA-induced knockdown of STAT3 decreased STAT3 levels in TSC2— cells. (C) RNA-Seq results show upregulated *IGF2* transcripts per kilobase million (TPM) in TSC2— cells transfected with either NT siRNA (Control) or STAT3 siRNA (siSTAT3). (D) STAT3 binding sites in human IGF2 and mouse Igf2 promoter regions. STAT3 enrichment in specific promoter regions of (E) human *IGF2* and (F) mouse *Igf2* genes was detected by ChIP-qPCR. Treatment of *Tsc2*^*-/-*^ MEFs with Stat3 inhibitor S3I-201 (100 nM for 18 hr) decreased Igf2 protein (G) expression as measured via Western blot and (H) secretion as measured via ELISA. (I) siRNA-mediated Stat3 knockdown also decreased Igf2 protein expression in *Tsc2*^*-/-*^ MEFs. (J) siRNA-mediated Igf2 knockdown decreased Stat3 phosphorylation but not total Stat3.

To investigate imprinting-independent IGF2 expression regulation, we considered a STAT3-dependent mechanism. STAT3, a known pro-oncogenic transcription factor [27] involved in LAM cell survival [28], was increased in TSC2— vs. TSC2++ LAM cells (Fig 3A). Human LAM cells with siRNA-mediated STAT3 knockdown (Fig 3B) had attenuated *IGF2* transcript levels (Fig 3C), suggesting that STAT3 may be a direct stimulator of *IGF2* transcription in TSC2-null cells. Next, we searched for STAT3 binding motifs in the human *IGF2* and murine *Igf2* gene promoter regions (P1-P5) and identified four: P2 and P4 in *IGF2*, and P2 and P5 in *Igf2* (Fig 3D). To test whether STAT3 bound to these specific genomic regions, we performed ChIP assays in *Tsc2*^*-/-*^ MEFs and TSC2— cells. We found that the predominant binding sites of STAT3 were in the *IGF2* P4 promoter region and the *Igf2* P2 promoter region (Fig 3E and 3F). Thus, STAT3 binds directly to *IGF2* promoter regions and may regulate *IGF2* expression in LAM.

To confirm that STAT3 mediates Igf2 up-regulation at the protein level, we measured Igf2 protein levels in cell lysates and Igf2 secretion of *Tsc2*^*-/-*^ MEFs that (1) were treated with STAT3 inhibitor VI (S3I-201) [29, 30] and (2) had knocked down Stat3 with specific siRNA. Igf2 protein expression was decreased in response to Stat3 inhibition (Fig 3G) and knock-down (Fig 3I). Treatment of *Tsc2*^*-/-*^ MEFs with STAT3 inhibitor S3I-201 also decreased Igf2 secretion (Fig 3H). While siRNA-mediated Igf2 knockdown inhibited Stat3 phosphorylation, it had little effect on total Stat3 levels (Fig 3J), suggesting that Igf2 activates Stat3. This data supports Stat3 as a regulator of Igf2 protein expression in *Tsc2*^*-/-*^ MEFs.

### Igf2 expression in *Tsc2*^*-/-*^ MEFs and IGF2 expression in TSC2— and primary LAM-derived cells are insensitive to rapamycin treatment

**Fig 4:**
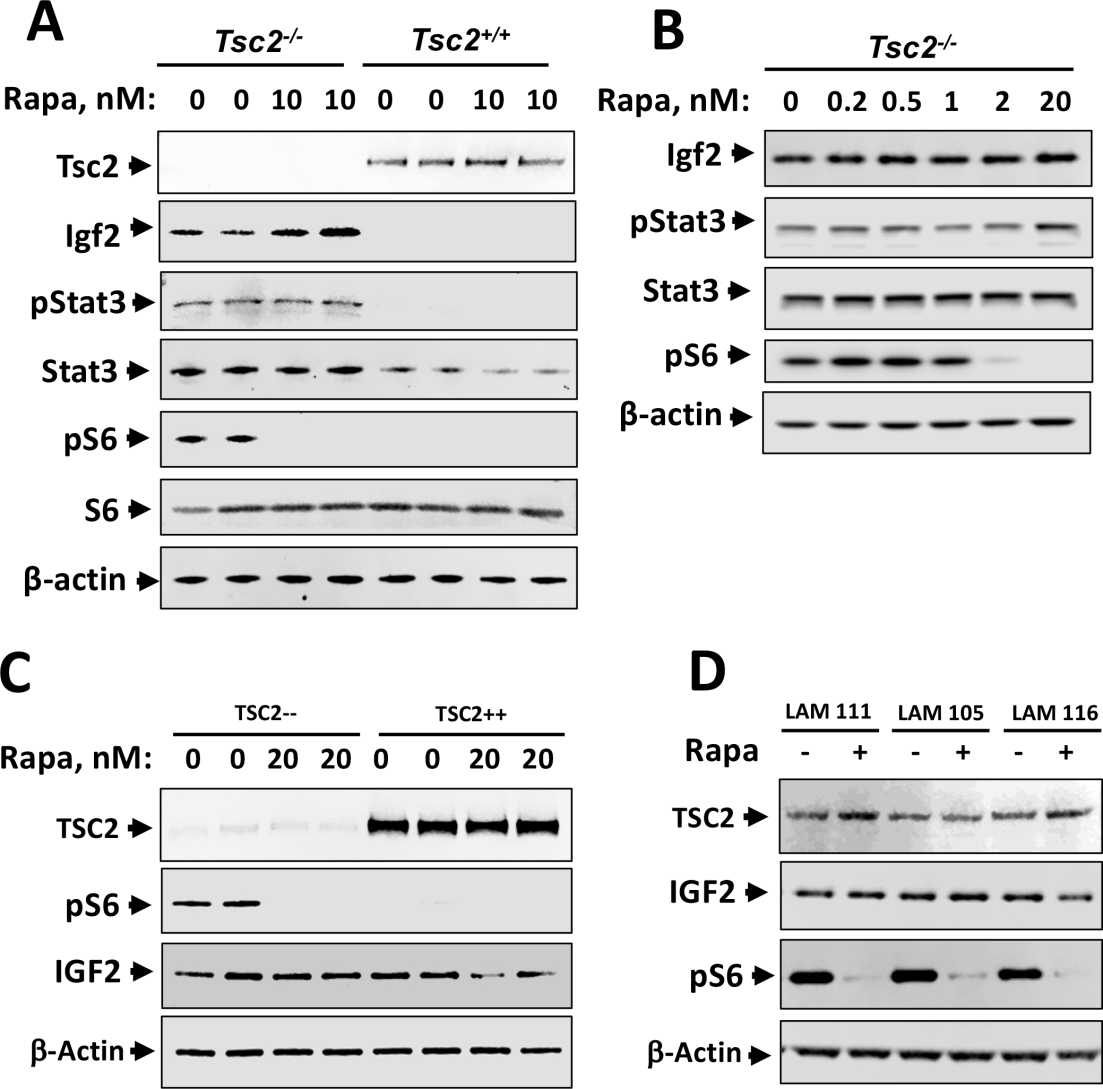
IGF2 expression is rapamycin-insensitive in *Tsc2*^*-/-*^ MEFs and human LAM cells. *Tsc2*^*-/-*^ and *Tsc2*^*+/+*^ MEFs were grown to near confluence, serum deprived for 2 hr and treated with indicated concentrations of rapamycin for 24 hr, followed by western blot analysis with indicated antibodies. (A) Treatment with 10nM rapamycin for 24 hr did not decrease Igf2 protein expression, although this dose completely suppressed pS6. (B) Igf2, Stat3, and pStat3 protein expression levels were unaffected by rapamycin treatment over a range of concentrations, while it completely inhibited pS6 at 2nM and 20nM concentrations. (C) IGF2 protein levels did not change in TSC2— and TSC2++ cells that were serum deprived for 2 hr and treated with 20nM rapamycin for 24 hr, as measured by western blot analysis. (D) IGF2 protein levels did not change in primary human LAM cells (LAM 111, LAM 105, LAM116) that were serum deprived for 2 hr and treated with 10nM rapamycin for 16 hr, as measured by western blot analysis. Images are representative of western blot analysis performed at least in three separate experiments.

To determine whether mTORC1 inhibition would affect intracellular Igf2 protein levels, *Tsc2*^*-/-*^ and *Tsc2*^*+/+*^ MEFs were treated with 10nM rapamycin. Consistent with Fig 2D, Igf2 was not observed in *Tsc2*^*+/+*^ MEFs (Fig 4A). Rapamycin treatment did not inhibit Igf2 in *Tsc2*^*-/-*^ MEFs, but pS6 protein was completely suppressed (Fig 4A). We also observed activation of Stat3 by analysis of phosphorylated Y705 Stat3 (pStat3) in *Tsc2*^*-/-*^ MEF lysates (Fig 4A). Next, we studied Igf2 expression in *Tsc2*^*-/-*^ MEFs over a range of rapamycin concentrations for 24 hours, and we found that while it completely inhibited pS6 at 2nM and 20nM concentrations, it had no effect on Stat3 levels and activation (Fig 4B).

Rapamycin treatment had no effect on IGF2 cellular protein level in TSC2— cells at a dosage that successfully suppressed pS6 (Fig 4C). Interestingly, in TSC2++ cells rapamycin appears to induce slight decrease in IGF2 levels (Fig 4C). Rapamycin treatment of three primary LAM-derived cell lines that expressed IGF2 found no change in protein expression levels compared to treatment with vehicle (Fig 4D). Thus, IGF2 protein expression in human LAM lung tissue and primary LAM lung cell lines was insensitive to rapamycin treatment.

## Discussion

The IGF2/IGF1/insulin pathway is a highly conserved evolutionary pathway regulating growth, aging and lifespan [31], and both IGF1 and IGF2 are regulators of mitogenic and metabolic signaling [13]. Our RNA-Seq and IHC results are consistent with a previous comprehensive immunohistochemical study that demonstrated significant expression of IGF2 in spindle-shaped lung LAM cells and modest expression of IGF1 and IGF1R, which is activated by both IGF1 and IGF2, throughout LAM lung tissue [15]. We found that *IGF2* has increased transcript expression in *Tsc2*^*-/-*^ MEFs and TSC2— cells derived from renal angiomyolipoma of a LAM patient. These expression differences extended to the protein level, as evidenced by increased expression and secretion of Igf2 in *Tsc2*^*-/-*^ MEFs compared to *Tsc2*^*+/+*^ MEFs, and presence of IGF2 in LAM102 TSC2— cells, as well as primary lung LAM cells. Knockdown of *Igf2* led to increased cell death and decreased cell viability in MEFs, and IGF2/Igf2 expression was not affected by rapamycin treatment.

Expression of *IGF2* is highly regulated [13]. In bone development and remodeling in the adult mouse, the *Igf2* P2 promoter regulates the fate of mesenchymal progenitors and regulates osteogenesis in a cell-autonomous and non-autonomous manner [32]. IGF2 expression is regulated by STAT3 during myogenic differentiation [33], and in LAM cells, the STAT3 pathway is important for survival [28]. Our results suggest that IGF2 expression is partially mediated via a STAT3 pathway that is rapamycin insensitive.

IGF2 is first translated as the 20 kDa preproIGF2 that after post-translational cleavage followed by glycosylation becomes proIGF2 [13]. The IGF2 we observed in MEFs and LAM cells is an approximately 20kDa protein that may be preproIGF2 or proIGF2. Proteases process this protein to the final 7.5 kDa IGF2 that is secreted and can be measured in circulation [13]. Mouse Igf2 is mainly expressed in fetal lung and turned off in the adult [13]. Human IGF2 is highly expressed during embryogenesis and its expression continues in adult lung, liver, brain and blood [13]. IGF2 has been shown to activate proliferation and inhibit apoptosis in multiple cell types [34]. Biallelic expression of *Igf2* induces tumor formation in p53 heterozygous mice, suggesting that increased Igf2 may suppress p53 tumor suppressor activity, and thus favor tumor development predominantly in females [35, 36]. In a leiomyoma, IGF2 was found to be the major driver of tumor growth [37], and IGF2 was found to be the most overexpressed gene in colorectal neoplasia [13] and highly expressed in mesenchymal tumors [38]. An intriguing study demonstrated that in each of 29 LAM lung samples studied, the mesenchymal chromatin binding protein HMGA2 was expressed along with IGF2BP2 (IMP2), a protein whose transcription is increased by HMGA2 and whose role is to stabilize *IGF2* mRNA [39]. Thus, it is likely IGF2 is involved in the autocrine and paracrine growth survival pathways with multiple feedback loops that are present in LAM cells.

Multiple tumors are characterized by loss of *IGF2* imprinting, which leads to increased IGF2 production, as both of its alleles become expressed [26]. However, we did not observe imprinting changes in renal LAM angiomyolipoma or LAM lung cells. Our findings are consistent with IGF2 expression in LAM being driven by transcription factors. Specifically, we found via a ChIP assay STAT3 binds to IGF2 promoter regions in human LAM and *Tsc2*^*-/-*^ MEFs.

There are six secreted binding proteins that bind to members of the insulin-like growth factor family [40]. In LAM lung tissue, four of these binding proteins (i.e., IGFBP2, IGFBP4, IGFBP5, IGFBP6) are expressed, with IGFBP2 having the highest levels [15]. Overexpression of IGFBP2 and IGFBP5 have been associated with poorer cancer prognosis [41]. A family of three RNA binding proteins (i.e., *Igf2bp1*, *Igf2bp2*, *Igfbp3*) that are mTOR Complex 2 (mTORC2) substrates are required for normal *Igf2* RNA splicing and for *Igf2* mRNA translation during mouse embryonic growth [42–44]. Our RNA-Seq results for *Tsc2*^*-/-*^ vs. *Tsc2*^*+/+*^ MEFs found that transcripts of several IGF binding proteins, including *Igfbp5*, were increased (S2 Table). Binding of IGF2 to IGFBP5 has been shown to enhance proliferative activity in some cells and decrease it in others [45]. Despite the large increase of *Igfbp5* transcripts we observed in *Tsc2*^*-/-*^ vs. *Tsc2*^*+/+*^ MEFs, there was minimal or no change in protein expression levels (data not shown). Published studies demonstrate that *Tsc2*^*-/-*^ MEFs secrete Igfbp5 which mediates mTORC1-dependent feedback inhibition of IGF-1 signaling [45]. IGFBP2 is expressed and colocalizes with estrogen receptor alpha in nuclei of LAM-patient derived cells, and its depletion in these cells results in decreased MAPK signaling, proliferation, migration and invasiveness, and increased apoptosis [46].

Overall, our results suggest that decreasing IGF2 concentrations in LAM tissues may serve as a therapy that is complementary to rapamycin treatment. Although IGF1R inhibitors exist, they have not performed well in oncology trials [47]. This could be due to incomplete targeting of all IGF1/IGF2 signaling, which is known to occur via alternative receptors. For example, IGF2 signals through IR-A in the leiomyosarcoma cell line SKUT1, which induces an autocrine feedback loop that enhances cell growth [47]. Rather than target receptor blockage, depletion of a ligand may be a more efficacious therapeutic alternative, as promising ongoing clinical trials with the IGF1/2 neutralizing antibody xentuzamab (BI836845) have shown thus far [48]. Specifically, in a phase Ib/II trial, the preliminary efficacy and clinical safety of BI836845 have been demonstrated when given in combination with everolimus and exemestane to patients with hormone receptor positive breast cancer [49]. Further research is necessary to determine if the IGF2 pathway in LAM is a driver of rapamycin-independent cell growth and whether this pathway is amenable to therapeutic intervention.

## Supporting information

S1 Fig Representative images of LAM lung lesions immunostained with control rabbit IgG (Upper panel) and rabbit anti-IGF2 antibody (lower panel). See Methods for Details.
(PDF)

S1 Table RNA-Seq metrics computed for quality control. The table contains the number of raw reads for the paired-end samples, the percentage of mapped reads among the total number of raw reads, the percentage of junction spanning reads among the mapped reads, the percentage of mapped bases that mapped to mRNA, and the mean insert size of mapped reads. Metrics are based on STAR alignment.
(XLSX)

S2 Table Top results for *Tsc2*^*-/-*^ vs. *Tsc2*^*+/+*^ MEFs comparison. All results with absolute fold change > 8 are included.
(XLSX)

S3 Table Top results for human TSC2—_vehicle vs. TSC2++_vehicle comparison. All results with absolute fold change > 8 are included.
(XLSX)

S4 Table Results of DAVID pathway analysis for differentially expressed genes from the *Tsc2*^*-/-*^ vs. *Tsc2*^*+/+*^ MEFs comparison. All results with enrichment scores > = 2 are included. The genes used in the pathway analysis had fold change > = 10 or fold change < = 0.1 in this comparison.
(XLSX)

S5 Table Results of DAVID pathway analysis for differentially expressed genes from the TSC2—_vehicle vs. TSC2++_vehicle comparison in human. All results with enrichment scores > = 2 are included. The genes used in the pathway analysis had fold change > = 8 or fold change < = 0.125 in this comparison.
(XLSX)

S6 Table Complete analysis of the methylation status of the imprinting control (IC1) region of the *IGF2* gene in human cell lines. (XLSX)
