# Supplementary material for: Rapamycin-independent IGF2 expression in Tsc2-null mouse embryo fibroblasts and human lymphangioleiomyomatosis cells
Source: PLoS One. 2018 May 14;13(5):e0197105. doi: 10.1371/journal.pone.0197105 (PMC5951544; doi:10.1371/journal.pone.0197105)
Supplement: S1 Fig — Representative images of LAM lung lesions immunostained with control rabbit IgG (Upper panel) and rabbit anti-IGF2 antibody (lower panel). See Methods for Details. (PDF) [file pone.0197105.s001.pdf]

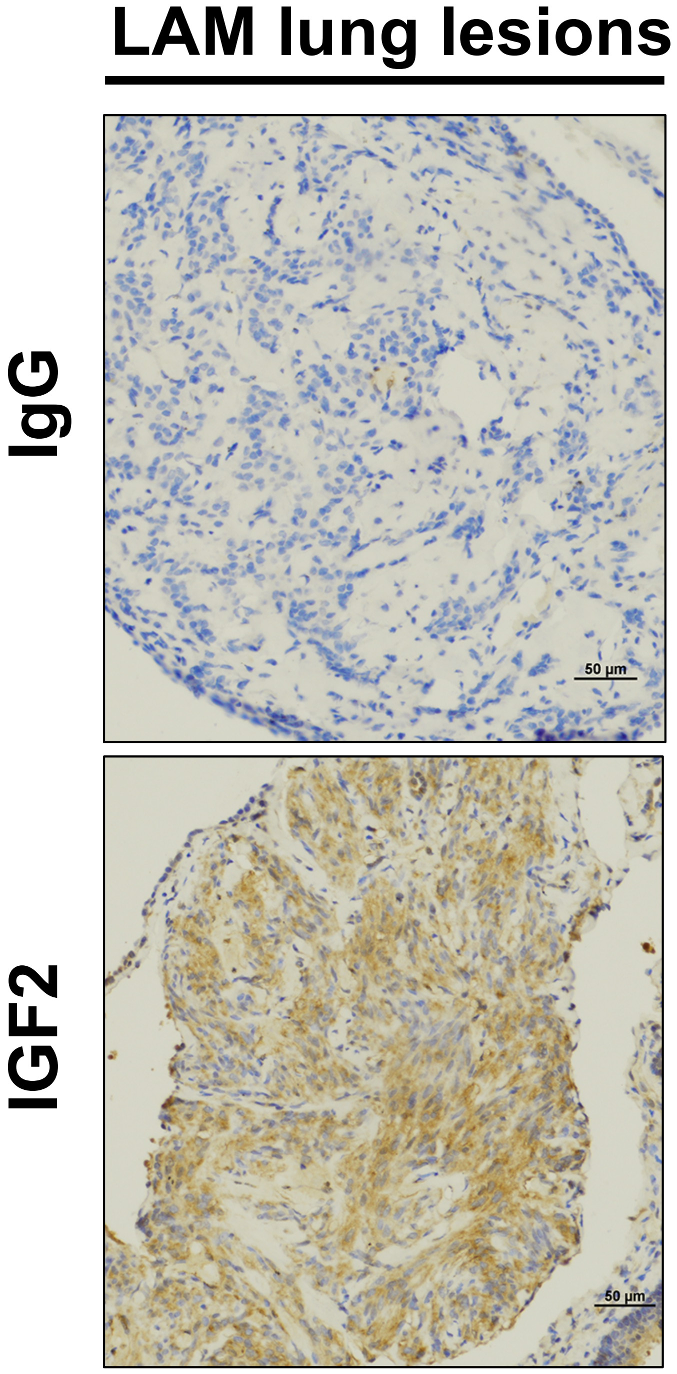

S1 Fig: Representative images of LAM lung lesions immunostained with control rabbit IgG (Upper panel) and rabbit anti-IGF2 antibody (Lower panel). See Methods for details.
